# Antibody transcytosis across brain endothelial-like cells occurs nonspecifically and independent of FcRn

**DOI:** 10.1038/s41598-020-60438-z

**Published:** 2020-02-28

**Authors:** John S. Ruano-Salguero, Kelvin H. Lee

**Affiliations:** 1 Department of Chemical and Biomolecular Engineering, University of Delaware, Newark, DE 19716 USA; 2 Delaware Biotechnology Institute, University of Delaware, Newark, DE 19711 USA

**Keywords:** Antibody therapy, Blood-brain barrier

## Abstract

The blood-brain barrier (BBB) hinders the brain delivery of therapeutic immunoglobulin γ (IgG) antibodies. Evidence suggests that IgG-specific processing occurs within the endothelium of the BBB, but any influence on transcytosis remains unclear. Here, involvement of the neonatal Fc receptor (FcRn), which mediates IgG recycling and transcytosis in peripheral endothelium, was investigated by evaluating the transcytosis of IgGs with native or reduced FcRn engagement across human induced pluripotent stem cell-derived brain endothelial-like cells. Despite differential trafficking, the permeability of all tested IgGs were comparable and remained constant irrespective of concentration or competition with excess IgG, suggesting IgG transcytosis occurs nonspecifically and originates from fluid-phase endocytosis. Comparison with the receptor-enhanced permeability of transferrin indicates that the phenomena observed for IgG is ubiquitous for most macromolecules. However, increased permeability was observed for macromolecules with biophysical properties known to engage alternative endocytosis mechanisms, highlighting the importance of biophysical characterizations in assessing transcytosis mechanisms.

## Introduction

The brain endothelial cells (BECs) that form the main structural component of the blood-brain barrier (BBB) are central to the protection of brain parenchyma. Unique from peripheral endothelium, BECs exhibit substantially reduced permeability of most bloodborne molecules^1^. Accordingly, this restrictive physiology also poses a formidable obstacle in the brain delivery of therapeutic molecules^2^. In particular, conventional immunoglobulin γ (IgG) antibody-based passive immunotherapies, which are structurally and functionally similar to endogenous IgGs, only demonstrate brain uptake of 0.1–0.3% of the injected dose^3,4^. Despite the need to improve the brain delivery of conventional therapeutic IgGs^5^, progress is hindered by the current lack of understanding regarding their interactions with BECs.

IgG-endothelium interactions in the periphery are dominated by the neonatal fragment crystallizable (Fc) receptor (FcRn). Following internalization of circulating IgG, the acidic microenvironment of endosomal compartments enables FcRn to bind and recycle IgG back to the lumen in a pH-dependent manner^6^. FcRn-mediated recycling therefore limits lysosomal degradation and contributes to the extended serum half-life of IgGs. However, FcRn can also mediate the abluminal transcytosis of IgG, which is exemplified in the transfer of maternal IgG across the placental endothelium. In this regard, FcRn is a unique transcytosis receptor, e.g. compared to the transferrin receptor (TfR)^7^, as it can shuttle its ligand bidirectionally to either cell surface (i.e. luminal recycling or abluminal transcytosis)^8^. Traditional transcytosis pathways are categorized as fluid-phase (e.g. macropinocytosis) or adsorptive-mediated, which occur via specific (e.g. receptor-mediated transcytosis (RMT)) or nonspecific (e.g. electrostatic adsorption) processes. The mechanisms regulating the preference for luminal or abluminal shuttling by FcRn remain to be determined, but are likely tissue and organ-specific^6,8,9^. Accordingly, FcRn functionality in BECs may not mirror those reported for other endo- or epithelium (e.g. intestinal) in which IgG transcytosis is observed^8^.

Since the initial detection of FcRn in BECs over 15 years ago^10^, its role in mediating recycling or transcytosis of IgG across BECs has remained uncertain. First, no study has yet confirmed the recycling of IgG by BECs *in vivo* or *in vitro*. Indirect evidence of potential FcRn-mediated recycling was provided by a recent *ex vivo* study demonstrating the partial localization of endogenous mouse IgGs within BECs to lysosomal compartments^11^. These findings have led to speculation whether lysosomal degradation is a contributing factor to the limited BBB permeability of IgG, but this hypothesis remains unconfirmed^11^. Second, there is no consensus on the role of FcRn in influencing the blood-to-brain transcytosis of IgG across BECs despite several notable studies^12–17^. The use of cross-species systems, e.g. human IgG in mice, has been identified as a potential confounding factor^18^ given the importance of binding affinity in proper FcRn-IgG engagement^19,20^. Similarly, FcRn knockout models may also generate confounding results as there is the potential to engage compensatory mechanisms that could mask the effect of FcRn deletion^21^. Alternative inhibitory and comparative studies, such as using excess levels of IgG or comparing IgGs with native or reduced FcRn binding^17^, have been identified as a more direct approach over FcRn knockout models^21^. These alternative IgG-based approaches can also investigate the potential role of other IgG-specific receptors in mediating IgG transcytosis across BECs, which may be commensurate with FcRn^12^.

An additional variable that may contribute to the conflicting findings is the influence of IgG concentration on the observed transcytosis pathway employed by BECs. Consistent with receptor-mediated processing, IgG transcytosis across BECs has been reported as being concentration dependent^18,22^. However, several reports observe no evidence of concentration-dependent phenomena^3,4,23^. A potential confounding factor between such findings is the presence or absence of endogenous IgG, which is influenced by the particular experimental method (e.g. *in situ* brain perfusion). The presence of endogenous IgGs could mask the influence of IgG-specific receptors on transcytosis, and result in concentration-independent observations. Accordingly, the decreasing serum concentration of exogenous IgG, which is exacerbated in FcRn knockout models or when the IgG lacks FcRn binding, and the presence of endogenous IgG represent experimental hurdles to the assessment of IgG transcytosis across BECs *in vivo*.

Here we investigated the role of FcRn or other IgG-specific receptors in influencing the transcytosis of IgG across BECs *in vitro* using BEC-like cells derived from human induced pluripotent stem cells (iBECs). We have previously shown that iBECs exhibit permeability of IgG that is more restrictive than that observed for the rat BBB *in vivo*^24–26^, which supports its use as a representative BBB model. To compare the influence of FcRn engagement we assessed the intracellular processing and transcytosis of IgGs lacking human FcRn recognition, and we used human IgG as a control, at varying concentrations and in the presence of excess human IgG. Our findings are consistent with FcRn as a key regulator of IgG recycling in BECs, help clarify the transcytosis mechanism influencing the limited BEC permeability of IgG, and exemplify the need to characterize biophysical attributes of macromolecules to meaningfully interpret BEC permeability.

## Results

### FcRn mediates IgG recycling in iBECs and reduces lysosomal accumulation

**Figure 1:**
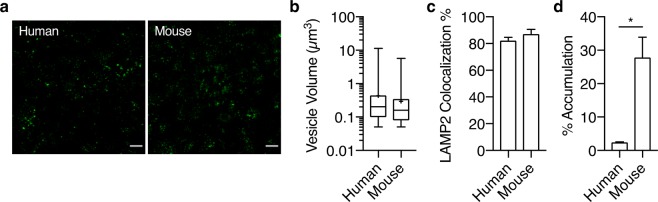
Lysosomal sorting of IgG is independent of FcRn-mediated salvaging in iBECs. (**a**) Representative deconvolved Airyscan super-resolution confocal images showing the intracellular vesicular structures containing fluorescently-labeled human or mouse IgG (green) in fixed iBECs after a 1-hr pulse with 667 nM of either IgG. Images are Z-projections based on average intensity, and scale bar represents 20 µm. (**b**) Representative vesicle diameter distribution of IgG-containing structures. Distributions are from one 192 × 192 µm^2^ image for each IgG, and are shown as boxplots with interquartile ranges and median. The mean is shown as a cross and error bars represent minimum and maximum values. (**c**) Quantification of colocalization between LAMP2 and either IgG using 3D object-based analysis. (**d**) Quantification of average vesicle intensity for either IgG relative to its inoculum intensity. Values in **(c)** and **(d)** represent means from three independent differentiations ± SEM, where each value is an average from five 192 × 192 µm^2^ images. Means were compared using two-tailed Student’s t-test (*P < 0.05).

To evaluate whether iBECs exhibit FcRn-mediated IgG recycling, we compared the intracellular processing of human IgG with that of mouse IgG, which lacks human FcRn recognition^20^. Initially we confirmed the expression of FcRn in iBECs via quantitative reverse transcription polymerase chain reaction (qRT-PCR) (Supplementary Fig. S1) and western analysis (Supplementary Fig. S2). Using high sensitivity super-resolution Airyscan confocal microscopy, intracellular visualization revealed no significant differences in the average size or number of vesicular structures that contained fluorescently-labeled mouse or human IgG after a one-hour pulse (Fig. 1a,b, and Supplementary Fig. S3). Immunocytochemistry of lysosomal-associated membrane protein 2 (LAMP2), a lysosomal marker, was performed because of the possibility of differences in lysosomal shuttling (Supplementary Fig. S4). Subsequent 3D object-based colocalization analysis revealed that over ~80% of mouse or human IgG-containing structures were LAMP2-positive lysosomes (Fig. 1c). LAMP2 colocalization analysis also revealed that there was no significant difference in their extent of lysosomal compartmentalization (Fig. 1c). Thus we hypothesized that although IgGs are eventually shuttled to any available lysosome, FcRn-mediated recycling of human IgG would lead to a lower extent of intracellular accumulation relative to mouse IgG. To quantify accumulation, the average vesicle intensity for each IgG was normalized by its corresponding inoculum solution intensity. Consistent with preferential recycling of human IgG by human FcRn, the normalized accumulation of human IgG was nearly 11-fold lower than that of mouse IgG (Fig. 1d).

### IgG transcytosis across iBECs is not receptor-mediated

**Figure 2:**
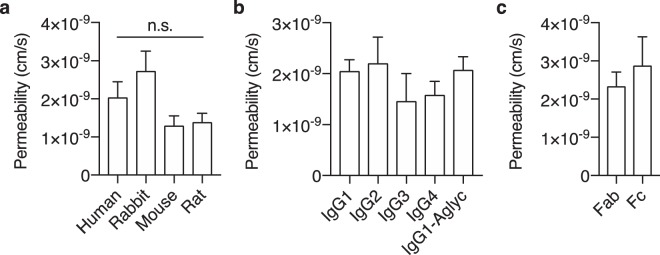
FcRn engagement does not alter iBEC permeability of IgG or its fragments. (**a**) The permeability of various serum-derived polyclonal IgGs of human, rabbit, mouse, and rat origin. (**b**) The permeability of the four human IgG subclasses, and an aglycosylated variant (IgG1-Aglyc). (**c**) The permeability of human IgG fragments, Fab and Fc. Molar concentration for all IgGs and fragments was 667 nM. Values are the mean of four (**a**,**b**) or three (**c**) independent differentiations ± SEM, and were compared using one-way ANOVA followed by Tukey’s multiple comparison test (n.s., P > 0.05) (**a** and **b**) or two-tailed Student’s t-test (**c**).

Given that FcRn was actively recycling human IgG, we evaluated whether FcRn was also mediating its transcytosis. To ensure the same intracellular phenomena were reproduced, the permeability of IgG was measured within the same one-hour timeframe and concentration previously used. Using live-cell microscopy-based permeability quantification, no significant differences between mouse and human IgG were observed (Fig. 2a). Similar permeability values were also observed for rat and rabbit IgG (Fig. 2a), which are comparable to mouse and human IgG with respect to human FcRn binding^20^, respectively. IgG from mice, rabbits, and rats also exhibit differences throughout the Fc domain that are distinct from the FcRn binding motif. It is therefore possible that these differences could engage other Fc-specific processes that alter their transcytosis by FcRn. Accordingly, the four human IgG subclasses (IgG1–4) were assessed separately as their Fc domains are conserved but only IgG3 exhibits a mutation on the FcRn binding motif^6^. Also, because glycosylation has been speculated to alter BEC permeability^27^, an aglycosylated IgG1 was assessed. Consistent with the initial observations, there was no significant difference in permeability between the four human IgG subclasses, or between glycosylated and aglycosylated IgG1 (Fig. 2b). Finally, any influence imparted by the Fc domain on IgG transcytosis was evaluated by comparing the permeability of the Fc with that of the antigen-binding fragment (Fab). In contrast with Fc-dependent transcytosis, the permeability between the two IgG fragments was not significantly different (Fig. 2c). Therefore these findings suggest the possibility that FcRn, or any other potential Fc receptor, do not directly mediate IgG transcytosis across iBECs.

### iBECs exhibit non-saturable transcytosis of IgG

**Figure 3:**
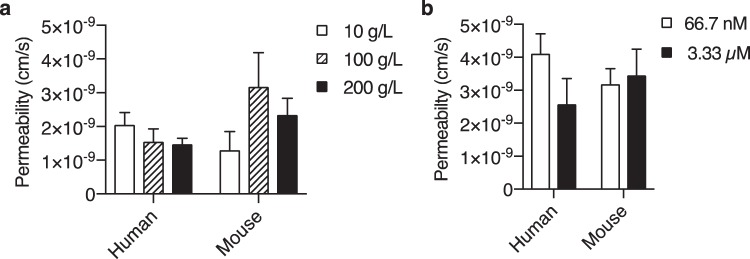
IgG transcytosis across iBECs is non-saturable regardless of FcRn engagement. (**a**) The permeability of human and mouse IgG at 667 nM in the presence of 10, 100, or 200 g/L of unlabeled human IgG, or (**b**) at 66.7 nM and 3.33 µM in the absence of unlabeled IgG. Values are the mean of three (**a**) or four (**b**) independent differentiations ± SEM, and were compared by two-way ANOVA followed by Sidak’s multiple comparison test to determine differences between groups or followed by Tukey’s multiple comparison test to determine differences within groups.

We next investigated whether the intracellular trafficking of IgG indirectly influenced its transcytosis. Processes tangential to transcytosis, such as FcRn-mediated recycling or lysosomal shuttling^11^, may divert the intracellular flux of IgG and lead to a decrease in permeability. FcRn-mediated recycling is concentration-dependent and has demonstrated enhanced recycling below 1600 nM in other endothelial cells *in vitro*^28^. Accordingly, the permeability of human and mouse IgG was evaluated at concentrations 10-fold lower (66.7 nM) and 5-fold higher (3.33 µM) than that previously tested. No evidence of altered permeability associated with FcRn-dependent shuttling was observed as both IgGs exhibited comparable values at low or high IgG concentrations (Fig. 3a). We also addressed whether any other IgG-specific mechanisms have the capability to saturate IgG transcytosis. To accomplish this, the permeability of labeled IgGs was measured in the presence of unlabeled human IgG at 1- to 20-fold endogenous serum levels in humans. The presence of any amount of unlabeled human IgG did not significantly alter the permeability of human or mouse IgG (Fig. 3b), which were again comparable. Based on the lack of concentration-dependent alterations in permeability at nanomolar concentrations or in the presence of unlabeled IgG, IgG transcytosis was not consistent with a saturable phenomenon. Consequently, the internalized amount of fluorescently-labeled IgG was also non-saturable and supports the notion that the endocytosis of IgG occurs via a nonspecific fluid-phase process.

### Characterization of saturable RMT of transferrin

**Figure 4:**
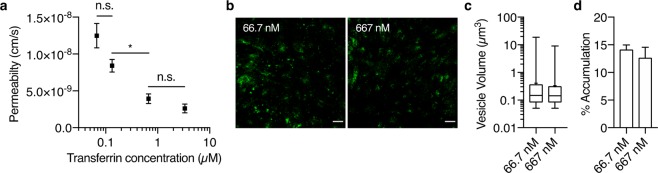
RMT of transferrin exhibits saturable kinetics and limited lysosomal shuttling in iBECs. (**a**) The permeability of transferrin at varying concentrations. (**b**) Representative deconvolved Airyscan super-resolution confocal images showing the intracellular vesicular structures containing fluorescently-labeled transferrin at 66.7 or 667 nM (green) in fixed iBECs after a 1-hr pulse. Images are Z-projections based on average intensity, and scale bar represents 20 µm. (**c**) Representative vesicle diameter distribution of transferrin-containing structures. Distributions are from one 192 × 192 µm^2^ image for each IgG and shown as boxplots with interquartile ranges and median. The mean is shown as a cross and error bars represent minimum and maximum values. (**d**) Quantification of average vesicle intensity for either concentration of transferrin relative to its inoculum intensity. Values in (**a**) are the mean from four independent differentiations ± SEM, and were compared using one-way ANOVA followed by Tukey’s multiple comparison test (n.s., P > 0.05, *P < 0.05). Values in (**c**,**d**) represent means from three independent differentiations ± SEM, where each value is an average from five 192 × 192 µm^2^ images, and were compared using two-tailed Student’s t-test.

As a comparison to the non-saturable mechanism observed for IgG, we assessed the well-characterized RMT of transferrin observed *in vivo*, but not currently reported for iBECs^29,30^. After confirming TfR expression in iBECs (Supplementary Fig. S1), the permeability of transferrin was evaluated as a function of concentration. Consistent with trends previously observed in literature, the permeability of transferrin decreased with increasing concentration until plateauing at micromolar concentrations (Fig. 4a). After confirming saturable RMT of transferrin across iBECs, we next investigated whether its intracellular processing was different from that observed for human or mouse IgGs. Additionally, we also addressed whether any differences exist between saturated (667 nM) and non-saturated (66.7 nM) transcytosis regimes for transferrin. Transferrin-containing vesicles exhibited a similar size distribution observed for IgGs, but were significantly more numerous regardless of transferrin concentration (Fig. 4b,c, and Supplementary Fig. S3a,b). Similarly, normalized accumulation analyses revealed comparable intracellular accumulation at either transcytosis regime (Fig. 4d). The accumulation metrics for transferrin were also significantly lower than those observed for mouse IgG (Supplementary Fig. 3c).

### Biophysical attributes of macromolecules influence iBEC permeability

**Figure 5:**
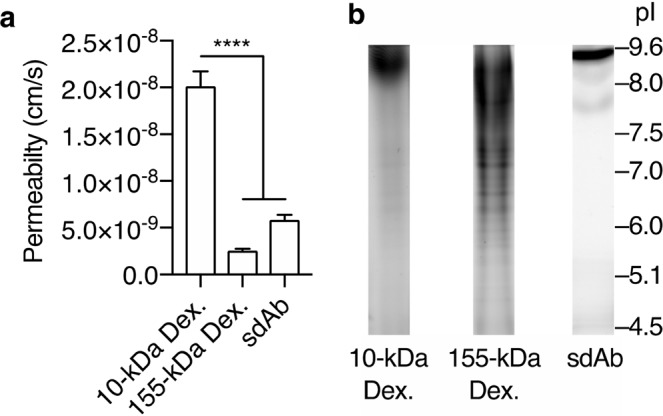
IEF reveals charge-dependent increase in iBEC permeability of a dextran and sdAb. (**a**) The permeability of 10-kDa and 155-kDa dextrans and a sdAb. (**b**) IEF images for the three macromolecules. Selected pI values from a concurrently run standard are shown on the right. Values in (a) is the mean from four independent differentiations ± SEM, and were compared using one-way ANOVA followed by Tukey’s multiple comparison test (****P < 0.0001). The lanes have been cropped from two images of a gel imaged using different excitation and emission settings (Supplementary Fig. S5).

The plateauing of transferrin permeability indicated that RMT was complemented by a transcytosis mechanism whose rate is concentration-independent, and therefore the amount transported is always proportional to the inoculum concentration. In agreement with other literature^31^, we hypothesized that this transcytosis mechanism occurred nonspecifically and originated from fluid-phase endocytosis. Such a mechanism would also explain the comparable permeability observed for IgG and its fragments, despite the 32–150 kDa molecular weight (MW) range. However, previous findings have demonstrated the potential for size-dependent differences in BBB permeability *in vivo*^24,32^. To investigate whether nonspecific transcytosis of macromolecules across iBECs is size-dependent, we assessed the permeability of 10-kDa dextran because it is above the size limit for paracellular transport at the BBB *in vivo*^33^ and would therefore be transported only by transcytosis. We found that the permeability of 10-kDa dextran was ~2.0 × 10^−8^ cm/s (Fig. 5a), nearly an order of magnitude higher than that observed for the 32 kDa Fc. Dextrans could engage specific processes in iBECs similar to that observed for other endothelial cells^34^, which may have contributed to the increased permeability. Thus, a 155-kDa dextran (which is of comparable size to IgG) was examined. The permeability of the 155-kDa dextran was significantly lower than that of the 10-kDa dextran (Fig. 5a) and suggested that dextran-specific interactions did not influence its transcytosis across iBECs. We next investigated whether the small size of 10-kDa dextran contributed to its increased permeability by assessing a comparably sized 14-kDa alpaca-derived single-domain antibody (sdAb), which targets a non-human antigen (green fluorescent protein). Despite only a 4 kDa difference in MW, the permeability of the sdAb was nearly 4-fold lower than that observed for the 10-kDa dextran (Fig. 5a). This finding suggested that size was likely not an important factor for the rapid transcytosis of 10-kDa dextran. Interestingly, comparing the permeabilities of the sdAb and larger macromolecules revealed a marginal, but significantly higher, permeability for the sdAb (Table 1). Enhanced permeability of certain sdAbs across the BBB *in vitro* has been reported previously and was attributed to highly positively charged isoelectric points (pI>9)^35^, which is hypothesized to foster adsorptive interactions with the negatively charged luminal surface of the BBB to facilitate transcytosis^7^. We performed isoelectric focusing (IEF) of the sdAb as well as 10- and 155-kDa dextrans to explore whether there were disparities in charge that might explain their enhanced permeability. Gel-based IEF revealed that both the 10-kDa dextran and sdAb exhibited a pI outside the standard range used (>9.6); whereas, the 155-kDa dextran exhibited a wide distribution that centered predominantly on a pI range of 7.0–9.6 (Fig. 5b). Accordingly, these observations support the role of charge in the increased transcytosis of both the sdAb and 10-kDa dextran as all the other macromolecules with lower permeability values exhibit a pI near or below the medium pH of 7.4 (Table 1).

**Table 1:** Summary of iBEC permeability values and select biophysical attributes of various macromolecules.

	Molecular Weight (Approximate)	Isoelectric Point	iBEC Permeability (×10^−9^ cm/s)	Abluminal Accumulation (% in 1 hour)	Multiple Comparisons
Human IgG	150 kDa	6.6–9.0^51^	2.24 ± 0.63	0.16	A
Mouse IgG	155 kDa	5.5–8.0^52^	1.30 ± 0.51	0.09	A
Human Fc	32 kDa	4.5–6.5^53^	2.34 ± 0.73	0.17	A
Human Fab	50 kDa	7.5–9.0^53^	2.89 ± 1.48	0.21	A
Human transferrin	80 kDa	5.2^54^	2.62 ± 1.21	0.19	A
10-kDa dextran	10 kDa	>9.6	20.30 ± 2.31	1.48	B
155-kDa dextran	155 kDa	7.0–9.6	2.50 ± 0.30	0.18	A
Alpaca sdAb	14 kDa	>9.6	5.85 ± 1.11	0.43	C

The pI of the dextrans (10 and 155 kDa) and sdAb are derived from Fig. 5b, and references for select pI values are noted. Permeability values are the mean from four unique differentiations, except for the Fab and Fc that were from three, ± SEM. The percentage of macromolecules initially added to the luminal compartment that accumulated in the abluminal compartment (after 1 hour) is provided as “Abluminal Accumulation (% in 1 hour)”. All analyte concentrations are 667 nM. The permeability values were compared using one-way ANOVA followed by Tukey’s multiple comparison test. Means within the same group are not significantly different (P > 0.05).

## Discussion

Currently, the mechanisms influencing the limited transcytosis of IgG across BECs remain unclear. Prevailing views suggest either a nonspecific or receptor-mediated mechanism, which often implicates FcRn given its ability to shuttle IgG bidirectionally to either cell surface. Our observations are consistent with a transcytosis mechanism that is independent of FcRn-mediated processes and does not exhibit a saturable phenomenon. Accordingly, we propose that IgG transcytosis across BECs occurs by a nonspecific process originating from fluid-phase endocytosis, in agreement with other conclusions including a recent report by our group demonstrating reduced IgG uptake via macropinocytosis inhibition^36,37^. To assess the influence of FcRn-mediated processing, we used a comparative IgG-based approach, similar to a previous *in vivo* report^17^, which exploits the well-characterized stringency of human FcRn to only bind a unique sequence present on the Fc domain of some IgGs^19,20^. Visualization and quantification of transcytosis within the same timeframe was achieved by using our previously developed *in vitro* BBB model that consists of a monolayer of iBECs on a COL1-based hydrogel^25^. We observed that despite significant differences in lysosomal accumulation that was consistent with preferential FcRn-mediated recycling^19^, all tested IgGs exhibited comparable iBEC permeability. As there was no evidence supporting RMT of IgG by FcRn, we assessed whether FcRn or any other IgG-specific receptor or mechanism could indirectly alter its permeability by performing concentration-dependent and inhibitory studies. The permeability of IgG was concentration-independent, even at levels near the dissociation constant of human FcRn^38^, and was also non-saturable regardless of its ability to engage FcRn. Thus, there was no apparent contribution from FcRn or other potential IgG receptors. Tripartite motif-containing protein 21 (TRIM21) was detected in iBECs (Supplementary Fig. S1), but it is known to mediate the ubiquitination of IgG-antigen complexes^39^ and no reports suggest it contributes to IgG transcytosis. The expression of TRIM21 remains to be confirmed *in vivo*, but it may be the unidentified IgG-associated protein previously reported in BECs because of its comparable MW^12^. Fc γ receptor IIb can also mediate the RMT of IgG^40^, however its expression was not detectable in iBECs (Supplementary Fig. S1) and remains unknown in BECs. Taken together, these results show that intracellular processes, including lysosomal degradation and FcRn-mediated recycling, do not indirectly influence the iBEC permeability of IgG.

An inherent complication of studies demonstrating saturable, or non-saturable, transcytosis of IgG across the BBB *in vivo* is the common practice of using cerebrospinal fluid (CSF) as a surrogate for the interstitial fluid (ISF) that surrounds brain parenchyma. Although CSF captures the levels of macromolecules in ISF via ISF-to-CSF convective-driven exchange^41^, it also captures the entry of macromolecules across the less restrictive (relative to the BBB^42^) endothelium and epithelium that forms the blood-CSF barrier (BCSFB). Thus, it is possible that any saturable transcytosis mechanism for IgG may be attributed to the BCSFB^43^, which also expresses FcRn and warrants further examination of its potential role^8^. However, an overlooked observation is that the steady-state CSF-to-serum levels of IgG3 in humans is at the same proportion as the other three IgG subclasses despite a lack of strong FcRn engagement^44^. Comparable CSF-to-serum levels between the human IgG subclasses support our conclusion that transcytosis across BECs is FcRn-independent, but also indicate that FcRn at the BCSFB may not significantly contribute to any preferential transcytosis at steady-state. Based on experimental limitations, the present study could not directly address the factors that influence brain-to-blood IgG transport across iBECs, which may include FcRn-mediated processes^12,13^.

Development of new approaches to increase the penetration of IgG or other macromolecules across the BBB can benefit from thorough *in vitro* characterization to appropriately interpret transcytosis phenomena. Our observation of saturable transcytosis of transferrin is consistent with other *in vivo* and *in vitro* reports also examining RMT by the TfR^45,46^, and the increased intracellular accumulation (relative to human IgG) supports the notion that lysosomal accumulation and degradation is a detriment to RMT^46^. Although TfR saturation in iBECs (~667 nM transferrin) is similar to that observed in mice (~500 nM of an anti-transferrin IgG^45^), saturation kinetics can vary from species to species^47^. Accordingly, evaluation of other RMT receptors should consider appropriate species-relevant models and potential saturation by native ligands at endogenous levels. As demonstrated here using Airyscan technology, the relatively low levels of transferrin and IgG accumulation suggest assessment of other transcytosis kinetics would also benefit from the added improvements in sensitivity and resolution facilitated by super-resolution confocal microscopy^46^. An important corollary of the saturation kinetics observed for transferrin is that any enhanced BEC permeability via RMT can eventually saturate and exhibit a nominal transcytosis rate. Because of the comparable permeability of antibodies (150 kDa), 155-kDa dextran, transferrin (80 kDa) at saturation, and Fab (~50 kDa) and Fc (~32 kDa) IgG fragments (Table 1), we propose that this nominal transcytosis rate likely originates from nonspecific fluid-phase endocytosis because it is constitutive and concentration-independent. Although other studies using dextrans of varying MW have presented evidence suggesting BBB permeability is size-dependent^24,32^, our observations for the sdAb (14 kDa) and 10-kDa dextran suggest charge is a more important determinant of BEC permeability. Dextrans derived from different bacterial sources or with varying chemical modifications may vary in charge and require thorough characterizations before interpreting BEC permeability given the importance of charge on BEC transcytosis^7^. Because the highly basic charges of the sdAb and 10-kDa dextran were indistinguishable using IEF, it is possible that their drastic differences in permeability are attributable to potentially small or large differences in pI^43^. Other biophysical attributes can also alter BBB permeability similar to that reported for cell-penetrating peptides^48^. Interestingly, dextrans with a MW 10 kDa or less can exhibit a random coil conformation^49^, which suggests that low MW dextrans may also influence BEC permeability via conformation. As charge and conformation-based alterations in the BBB permeability of macromolecules may not necessarily exhibit saturable phenomena^48^, a lack of *a priori* biophysical characterization may lead to misclassifications of transcytosis mechanisms.

In summary, our findings demonstrate that IgG transcytosis across an *in vitro* BBB exhibits a non-saturable and nonspecific mechanism, and supports the use of RMT approaches or modifications of biophysical properties, such as pI, to achieve improved brain uptake of therapeutic IgGs^7^. Additionally, this study also supports the use of *in vitro* BBB models, in combination with thorough biophysical characterizations, to provide useful assessment of therapeutic candidates before translation to preclinical models.

## Methods

### Hydrogel-based *in vitro* BBB model

COL1 from rat tendon (Corning) was prepared as a hydrogel on 8-chambered glass coverslips based on methods previously described^25^. IMR90-4 (WiCell) human induced pluripotent stem cells were maintained and differentiated to BECs as previously described^25,26,50^. After 8 days of differentiation, cells were dissociated with STEMPRO Accutase (Life Technologies) and suspended as single-cells. Cells were then added to preassembled hydrogels at a density of at least 1 million cells/cm^2^. The cells were allowed to form a confluent monolayer by replacing the medium, which consisting of 0.1% human serum from platelet poor plasma (MilliporeSigma) in human endothelial SFM (Life Technologies), for the next two subsequent days. Construction of the hydrogel-based BBB model is illustrated in the supplement (Supplementary Fig. S6a). All experiments were performed on the second day after subculture.

### Quantitative reverse transcription polymerase chain reaction

Confluent monolayers of iBECs were dissociated with STEMPRO Accutase and pelletized. Total RNA was isolated from the cell pellet using the RNeasy Micro Kit (Qiagen). qRT-PCR was performed with the TaqMan RNA-to-Ct 1-Step Kit (Applied Biosystems) using commercial primer/probe sets (Integrated DNA Technologies). The total RNA from three independent differentiations was used for qRT-PCR analysis and run in technical triplicate.

### Western analysis of FcRn

Frozen, pelletized iBECs were lysed with radioimmunoprecipitation assay (RIPA) buffer at 0.5 million cells/mL and boiled at 95 °C for 10 mins in sodium dodecyl sulfate (SDS) loading buffer (New England BioLabs) containing dithiothreitol. Biotinylated human FcRn (ACRO Biosystems) was loaded at 0.5 µg per lane and used as a positive control. Gel electrophoresis was performed in precast 4–20% Mini PROTEAN TGX gels (Bio-Rad), and the proteins were then transferred to Immobilon-P membranes (EMD Millipore). The membrane was blocked with 3% non-fat dry milk in Tris-buffered saline with TWEEN-20 (3%-TBST, Sigma) and then probed overnight at 4 °C with 2 µg/mL anti-human FcRn antibody in 3%-TBST (sc-66892, Santa Cruz Biotechnology). The membrane was then probed with alkaline phosphatase-conjugated goat anti-rabbit antibody at a 1:5000 dilution (Sigma) for 1 hour at 4 °C. Signals were detected with an enhanced chemifluorescence substrate (GE Healthcare Life Sciences) and imaged using a Typhoon FLA-7000 scanner (GE Healthcare Life Sciences).

### Fluorophore-IgG conjugation and deglycosylation

Pooled human IgG (Gammagard) and the four human IgG subclasses (MilliporeSigma) were dialyzed in PBS to remove glycine in the storage formulation. Mouse, rat, and rabbit IgGs (MilliporeSigma) were obtained as a powder and reconstituted in PBS. Fluorescent labeling of IgG was performed using a 2 mg-scale Alexa Fluor 647 Protein Labeling Kit (Invitrogen) according to the manufacturer’s protocol. Fluorescently-labeled IgG1 was deglycosylated using a 0.5 mg-scale GlycINATOR spin column (Genovis) according to the manufacturer’s protocol. All labeled IgGs were concentrated using a spin column with a MW cutoff of 2.5 kDa to achieve a concentration above 5 g/L.

### Pulse-chase assay and immunocytochemistry

After iBECs were pulsed with Alexa Fluor 647-labeled IgG (human or mouse, 667 nM) or human transferrin (66.7 and 667 nM, Jackson ImmunoResearch) diluted in Phenol-free Ham’s F-12 (Caisson) for 1 hour, the fluorogenic solutions were replaced with Phenol-free Ham’s F-12. Immediately after, 16% (v/v) paraformaldehyde (Electron Microscopy Sciences) was added to the medium for a final concentration of 4% (v/v). Following 30 minutes of fixation, cells were extensively rinsed with PBS. Cells were then permeabilized with 0.1% (v/v) Triton X-100 (MilliporeSigma) for 5 minutes, treated with 3 µg/mL mouse anti-LAMP2 antibody (Invitrogen) overnight at 4 °C, and then extensively rinsed with PBS. For detection, the cells were then treated with 5 µg/mL Alexa Fluor 488-labeled rabbit anti-mouse IgG (Invitrogen) for 1 hour at room temperature. Nuclei were labeled with NucBlue (DAPI; Life Technologies) as recommended by the manufacturer.

### Super-resolution Airyscan confocal microscopy

Fixed samples were imaged with a Zeiss 880 confocal microscope equipped with an Airyscan detector and a C-Apochromat 40× water immersion objective (NA 1.2) (Zeiss). Images with a voxel size of 0.0497 × 0.0497 × 0.083 µm^3^ (x-y-z, 3876 × 3876 × 1 pixel) were captured using the Airyscan Super-Resolution mode in Zen Black (Zeiss) and an appropriate filter cube. An optimal laser and gain setting was determined for each sample (e.g. 66.7 nM human transferrin), and was not changed when imaging biological replicates. The same imaging parameters were used to generate standard curves for images obtained from each serially diluted inoculum solution. Accordingly, all corresponding images for each sample, including the standard solution images, were batch processed in Zen Black using the same deconvolution parameters.

### Quantitative image processing and analysis

Object-based statistics and 3D object-based colocalization was performed in Volocity (Perkin Elmer). First, objects were detected using the ‘Find Object’ function for each z-stack image. Threshold intensity values were set as 3 standard deviations from the mean for each image. Large overlapping objects were segmented using the ‘Separate Touching Object’ function with a suggest volume size of 0.5 µm^3^. Objects with a volume less than 0.05 µm^3^ were excluded from all analysis as they were below the resolution of the images. For colocalization analysis, the ‘Calculate Object Colocalization’ function was used to measure the Manders M1 and M2 overlap coefficient between the Alexa Fluor 488 (LAMP2) channel and the Alexa Fluor 647 (macromolecule) channel. Colocalized objects with less than 30% overlap (M1 or M2 < 0.3) were not considered colocalization events in our analysis. Colocalization is represented as the percentage of vesicles that colocalized with LAMP2 relative to the total number of vesicles.

### Live-cell permeability assay

After medium replacement on the second day post-subculture, solutions containing fluorescent macromolecules and internal control were prepared. Unless noted otherwise, medium was replaced with 667 nM of fluorescently-labeled macromolecules in Phenol-free Ham’s F-12. As an internal control, each solution also contained 2 µM sodium fluorescein, except for 155-kDa dextran which used a Alexa Fluor 647-labeled human IgG as an internal control. Datasets with internal controls that exhibited permeability values 2-fold higher than that previously reported were not analyzed because they were indicative of a non-confluent iBEC monolayer^25^. After aspiration of the culture medium, the analyte-containing solutions were added to each chamber of the 8-chambered glass coverslip. The vessels were then immediately mounted in a preheated stage within a humidified enclosure at 37 °C and 5% CO_2_. Imaging was performed with a Zeiss 710 confocal microscope with a C-Apochromat 40× water immersion objective (NA 1.2). Time-lapse z-stacks that contained both the luminal and abluminal (hydrogel) compartments were captured every 15 minutes using image acquisition parameters previously described^25^. Briefly, time-lapse z-stacks were processed in ImageJ as previously described and the average intensity of the fluorescent analyte within the hydrogel was measured at each time point^25^. The apparent cellular permeability, P_app_, was calculated using the equation

$${P}_{app}=\frac{V}{A\cdot {I}_{solution}}\frac{d{I}_{hydrogel}}{dt}$$

where V is the volume of the hydrogel, A is the lateral area of the chamber area, I_solution_ is the intensity of the luminal solution and dI_hydrogel_/dt is the slope determined from the intensity versus time data measured via ImageJ. An illustration of the permeability measurement procedure is provided in the supplement (Supplementary Fig. S6b). The 10-kDa dextran (labeled with Texas Red) was purchased from Invitrogen (D1828), the 155-kDa dextran (labeled with tetramethylrhodamine) was purchased from MilliporeSigma (T1287), and the anti-green fluorescent protein sdAb (labeled with ATTO 647 N) was purchased from Chromotek. Human Fab and Fc (labeled with Alexa Fluor 647) was purchased from Jackson ImmunoResearch Laboratories.

### Isoelectric focusing

Polyacrylamide gel-based 1D IEF was performed using a pH 3–10 Criterion IEF Precast Gel (Bio-Rad). Samples were loaded at approximately 2 µg, and the 4.45–9.6 pI standard (Bio-RaD) was diluted 5-fold. The gel was run in a Criterion cell (Bio-Rad) according to the manufacturer’s protocol. Afterwards, the pI standard was visualized with SYPRO Ruby (Invitrogen) using the ‘Rapid’ protocol provided by the manufacturer.

### Statistical analysis

GraphPad Prism 7 was used for statistical analysis. Comparison of data was performed using Student’s t-test, and one-way and two-way ANOVA with α = 0.05. All experiments were carried out with at least three biological replicates, where a unique differentiation (derived from an independent passage) constitutes one biological replicate, to determine statistical significance. The selection of appropriate statistical test and exact sample size is indicated in each figure legend.

## Supplementary information

Supplementary Information.
