# Supplementary material for: Antibody transcytosis across brain endothelial-like cells occurs nonspecifically and independent of FcRn
Source: Sci Rep. 2020 Feb 28;10:3685. doi: 10.1038/s41598-020-60438-z (PMC7048754; doi:10.1038/s41598-020-60438-z)
Supplement: Supplementary file 1 — Supplementary Information. [file 41598_2020_60438_MOESM1_ESM.docx]

**SUPPLEMENTARY INFORMATION**

Antibody transcytosis across brain endothelial-like cells occurs nonspecifically and independent of FcRn

John S. Ruano-Salguero,^1,2^ Kelvin H. Lee^1,2,*^

^1^Department of Chemical and Biomolecular Engineering, University of Delaware, Newark, DE 19716, USA.

^2^Delaware Biotechnology Institute, University of Delaware, Newark, DE 19711, USA.

^*^Correspondence to KHL@udel.edu

**Supplementary Figure S1.** Evaluation of gene expression by qRT-PCR.

**Supplementary Figure S2.** Protein expression of FcRn by western analysis.

**Supplementary Figure S3.** Differential intracellular processing of IgG and transferrin by iBECs after 1 hour.

**Supplementary Figure S4.** Visualization of LAMP2 colocalization in iBECs after 1 hour.

**Supplementary Figure S5.** Fluorescent images of a single IEF gel.

**Supplementary Figure S6.** Illustration of hydrogel-based BBB model.

**Supplementary Figure S1.** Evaluation of gene expression by qRT-PCR. Levels of mRNA for each gene are shown relative to GAPDH (reference gene). Values are from three independent differentiations, run in technical triplicate, ± SEM.

**Supplementary Figure S2.** Protein expression of FcRn by western analysis. The left lane is purified human FcRn (biotinylated) and the right lane is iBEC lysate.

**Supplementary Figure S3.** Differential intracellular processing of IgG and transferrin by iBECs after 1 hour. (**a**) Volume and (**b**) number of vesicles containing fluorescently-labeled macromolecules. (**c**) Quantification of average vesicle intensity relative to inoculum intensity. Values represent the means from three independent differentiations ± SEM, where each value is an average from five 192 × 192 µm^2^ images. Means were compared using one-way ANOVA followed by Tukey’s multiple comparison test (*P < 0.05, **P < 0.01).

**Supplementary Figure S4.** Visualization of LAMP2 colocalization in iBECs after 1 hour. Representative deconvolved Airyscan super-resolution confocal images showing the intracellular vesicular structures containing fluorescently-labeled (**a**) human or (**b**) mouse IgG (green) and LAMP2-positive lysosomes (red). Images are Z-projections based on average intensity, nucleus is stained with DAPI (blue), and scale bar represents 10 µm.


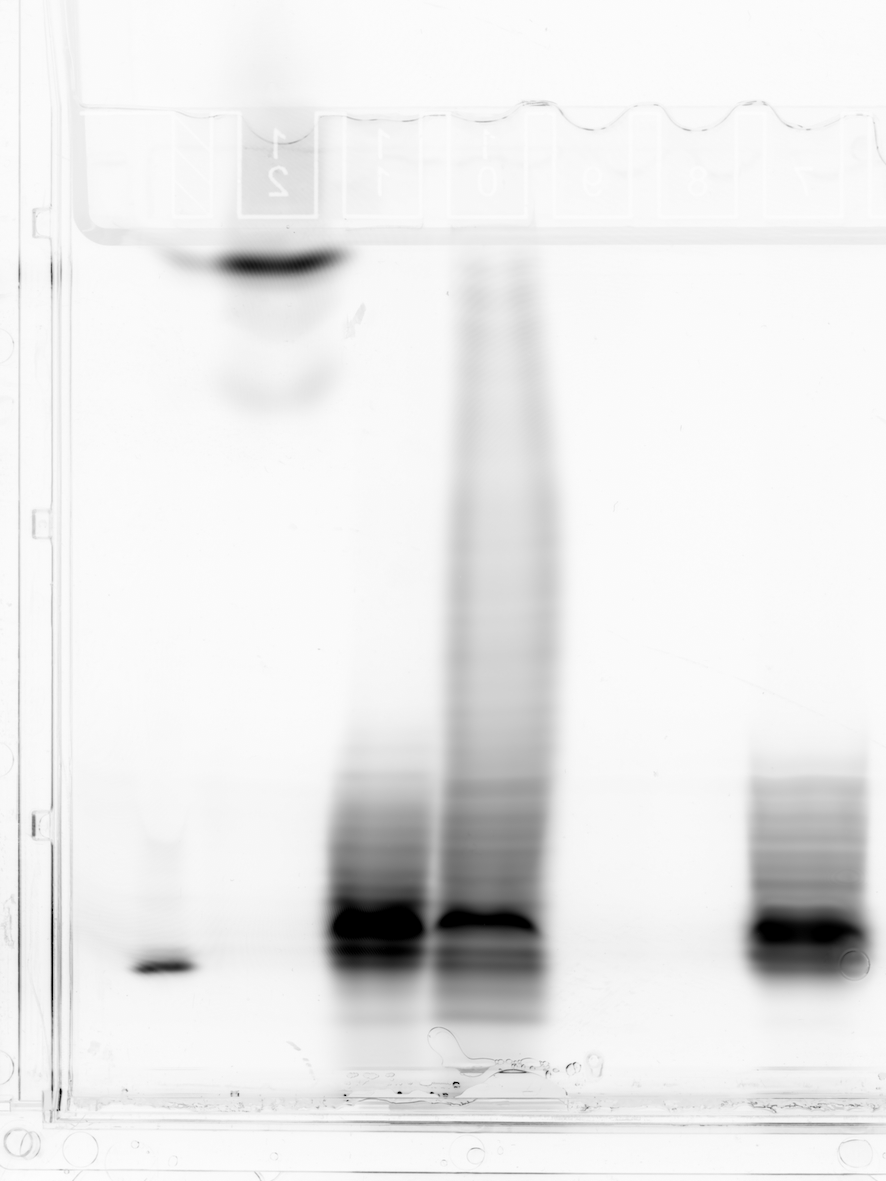

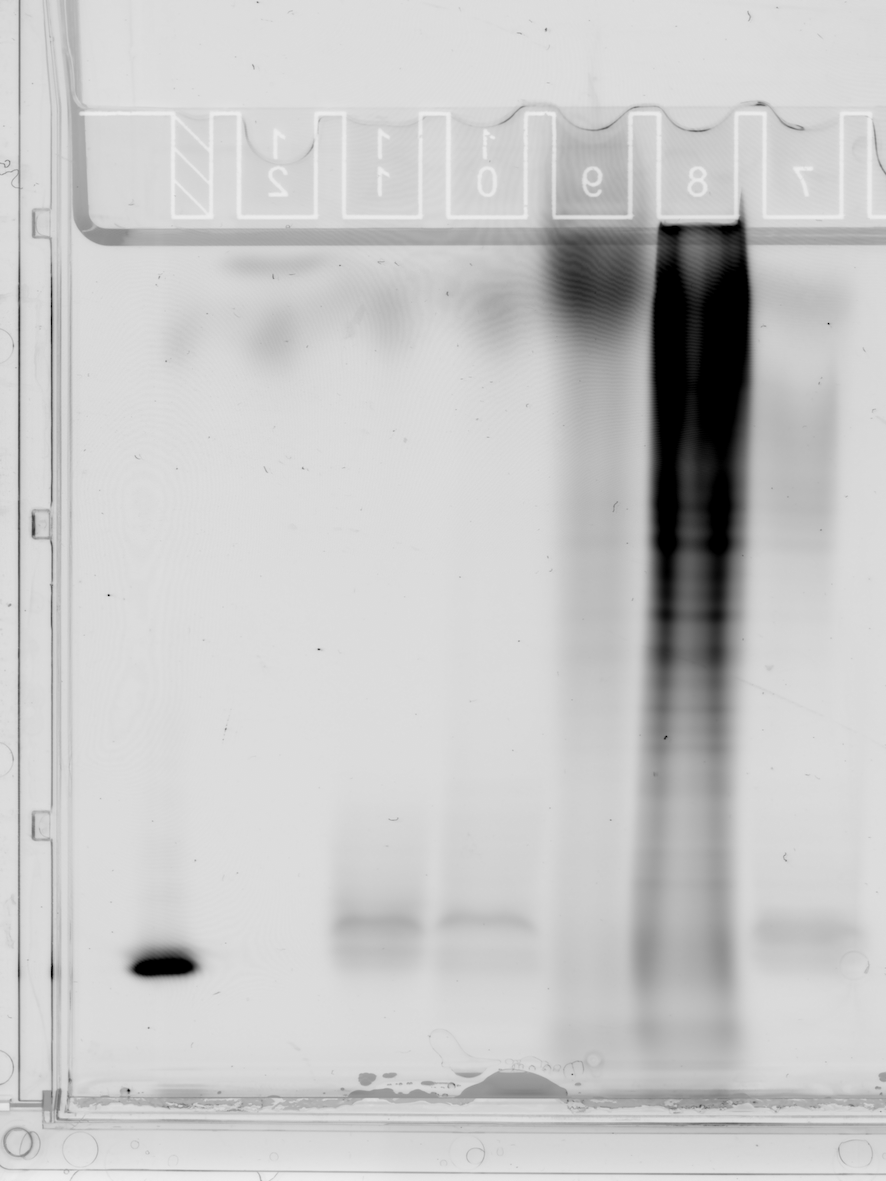


**Supplementary Figure S5.** Fluorescent images of a single IEF gel. The excitation/emission wavelengths were 635/670 nm (left) and 532/580 nm (right). The IEF standard is denoted †, the sdAb is denoted ††, and the 10-kDa and 155-kDa dextrans are denoted * and **, respectively.


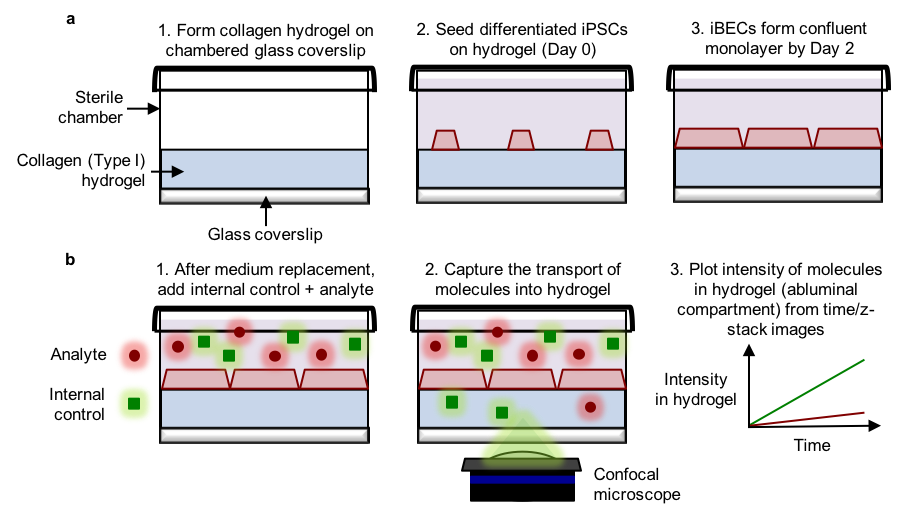


**Supplementary Figure S6.** Illustration of hydrogel-based BBB model. (**a**) Procedure to form a confluent iBEC monolayer on a collagen type I hydrogel. (**b**) Measurement and analysis of monolayer permeability.
